# Assisted full-endoscopic spine surgery for lumbar spinal stenosis: Technical note and learning curve

**DOI:** 10.1016/j.bas.2026.105935

**Published:** 2026-01-11

**Authors:** Koichiro Ono, Daisuke Fukuhara, Yuka Yamami, Yushi Yamaguchi, Kazuma Miura, Yuki Kasuga, Kaichi Sato, Satoshi Takamoto, Naoya Takabayshi, Hiroshi Kawaguchi, Makoto Hirao

**Affiliations:** a Department of Orthopedic Surgery, Nippon Medical School, Tokyo, Japan; b Department of Orthopaedics, Graduate School of Medical Science, Kyoto Prefectural University of Medicine, Japan

**Keywords:** Assisted full-endoscopic spine surgery, AFESS, Lumbar spinal stenosis, Minimally invasive decompression, Learning curve, Facet preservation

## Abstract

**Introduction:**

Lumbar spinal stenosis (LSS) causes disabling back and radicular pain. Full-endoscopic spine surgery offers minimally invasive decompression, but uniportal and biportal approaches have limitations. Assisted full-endoscopic spine surgery (AFESS) combines the advantages of both techniques to enhance decompression while preserving facet joints.

**Research question:**

Can AFESS provide effective decompression with high facet preservation for LSS, and what is the learning curve for this technique?

**Material and methods:**

We retrospectively reviewed 33 patients with single-level LSS treated with AFESS, divided into initial (20 cases) and advanced (13 cases) phases. Outcomes assessed at a minimum 6-month follow-up included operative time, facet joint preservation ratio, complications, and visual analog scale (VAS) scores for back and leg pain.

**Results:**

Operative time decreased significantly from the initial to the advanced phase (mean 99 vs 72 min; p < 0.01). Facet joints were more preserved in the advanced phase (76.2 % vs 68.9 %, p < 0.05). In the initial and advanced phases, mean lumbar VAS improved from 4.2 to 1.1 cm and 2.8 to 0.2 cm, respectively. Radicular VAS improved from 7.7 to 0.8 cm (initial) and 6.9 to 0.9 cm (advanced) (p < 0.01 for all). Two patients (6 %) experienced minor complications in the initial phase, with no permanent deficits.

**Discussion and conclusion:**

AFESS is safe and effective, achieving adequate decompression with high facet preservation and significant pain relief in LSS patients. By integrating uniportal and biportal endoscopic approaches, AFESS overcomes limitations of each. The well-defined learning curve suggests it can be efficiently incorporated into clinical practice with minimal complications.

## Highlights

• **Assisted Full-Endoscopic Spine Surgery (AFESS) is a modified biportal technique that integrates uniportal high-definition optics with independent instrument manipulation.**
• **The technique demonstrated a clear learning curve, with mean operative time significantly decreasing from 99 min in the initial phase to 72 min in the advanced phase.**
• **Facet joint preservation significantly improved from 68.9 % in the initial phase to 76.2 % in the advanced phase, achieved through inside-out decompression.**
• **Significant pain relief was achieved in both phases, with final mean radicular VAS scores dropping to less than 1.0 cm in both groups.**
• **AFESS showed a favorable safety profile with a low complication rate (6 %) and no permanent neurological deficits.**

## 1 Introduction

Lumbar spinal stenosis (LSS) is a prevalent degenerative spine condition and a leading cause of pain and disability in older adults, representing the most frequent indication for spine surgery in this population (Walter and O'Toole, 2022; Hermansen et al., 2022). Traditionally, symptomatic LSS has been managed with open decompressive laminectomy, considered the gold-standard surgical treatment (Kwan et al., 2019). Over the past decade, however, the pursuit of less invasive interventions has driven significant evolution in minimally invasive spine surgery, with endoscopic techniques introduced to achieve adequate neural decompression while minimizing iatrogenic tissue injury (Sharma et al., 2025; Kwon et al., 2023).

Full-endoscopic spine surgery offers distinct advantages compared to open procedures, including minimal paraspinal muscle dissection, reduced intraoperative bleeding, shorter hospital stays, and accelerated postoperative recovery (Komp et al., 2015; Yamaguchi, 2024; Yang et al., 2023). With advancements in endoscopic optics and instrumentation, full-endoscopic spine surgery (FESS) has expanded to treat various degenerative conditions, including central lumbar stenosis. Recent clinical studies have demonstrated that endoscopic decompression for LSS can achieve outcomes comparable to those of conventional surgery, while conferring significant perioperative advantages (Yang et al., 2023; Lee et al., 2024a).

Despite its promise, full-endoscopic lumbar decompression remains technically demanding, with widespread adoption tempered by a relatively steep learning curve, particularly during the early adoption phase (Chung et al., 2025). Surgeons must adapt to a two-dimensional endoscopic view of a three-dimensional operative field and develop precise hand-eye coordination with specialized instruments. During the initial learning phase, endoscopic procedures typically require longer operative times and present higher technical difficulty, which has been associated with an increased risk of complications such as recurrent herniation or dural tears (Yorimitsu et al., 2022; Park et al., 2022). The constraints of working through a single narrow endoscopic channel—where only one instrument can be used at a time—further contribute to this challenge (Yeung, 2022a).

Some surgeons perceive that conventional uniportal full-endoscopic approaches for lumbar stenosis may impose practical constraints related to the single working corridor rather than to the optical system itself. Although modern endoscopes can be translated and rotated and used with angled lenses to visualize different regions of the surgical field, the requirement to perform all maneuvers through a single portal can make instrument handling and ergonomics challenging, especially during the learning phase. In addition, because only one instrument can usually be used alongside the endoscope, opportunities for true bimanual work are limited, which may reduce fine control compared with techniques that use separate camera and working portals (Chung et al., 2025; Yeung, 2022a).

Unilateral biportal endoscopic (UBE) techniques were developed as an alternative configuration that employs two small incisions, one primarily for the endoscope and the other for working instruments. This arrangement creates a bimanual working environment and allows independent manipulation of the endoscope and instruments, which many surgeons experience as more similar to conventional microscopic decompression (Choi and Kim, 2019). Clinical series of UBE for lumbar decompression have reported adequate neural decompression with minimal tissue disruption and favorable perioperative recovery profiles (Park et al., 2023; Lee et al., 2024b).

Building on these advancements, we describe Assisted Full-Endoscopic Spine Surgery (AFESS) for lumbar spinal stenosis, a modified unilateral biportal endoscopic technique in which a monoportal full-endoscopic system is inserted through a camera portal and conventional spinal instruments are introduced through a separate working portal (Kaneko et al., 2023; Chien et al., 2022). Rather than representing a completely new paradigm or the “best of both worlds” between uniportal and biportal surgery, AFESS can be regarded as a variation of the biportal concept that incorporates a full-endoscopic system into one portal while maintaining a two-portal working configuration. Initial reports have suggested that AFESS may permit bilateral decompression with high facet preservation, but published experience remains limited (Kaneko et al., 2023; Chien et al., 2022).

The primary aim of this technical note is to describe the AFESS procedure in detail and to report our early experience with its learning curve in patients with single-level lumbar spinal stenosis. We present the stepwise surgical technique and a phase-based analysis of operative time across consecutive cases as a descriptive assessment of how procedural efficiency evolved during the introduction of AFESS in our practice. By documenting these technical steps and early learning observations, we intend to provide practical information for surgeons considering adopting this modified biportal endoscopic approach, while acknowledging that its comparative effectiveness relative to existing uniportal and biportal techniques remains to be established.

## 2 Materials and methods

Study Design and Patient Selection We retrospectively analyzed 33 consecutive patients who underwent AFESS for single-level lumbar spinal stenosis between June 1, 2023, and September 2024 (Table 1). All procedures were performed at a single institution by one fellowship-trained spine surgeon (K.O.). Patients were stratified into two chronological cohorts to evaluate the learning curve and the impact of technical refinements.

**Table 1:** Patient demographics and clinical data.

Characteristic	Initial phase	Advanced phase	*p* value
Case number	20	13	–
Age (range)	67.3 (23–87)	63 (41–83)	*n.s.*
Sex (Male/Female)	6/14	3/10	*n.s.*
Surgical levels (L2/3,3/4,4/5,5/S1)	2, 6, 12, 0	2, 3, 6, 2	*n.s.*
Estimated blood loss (mL)	Minimal	Minimal	*n.s.*
Postoperative hospitalization time (days)	5.4	3.5	*p* = *0.026*
Follow-up periods (month)	18.6	7.7	*p < 0.01*
Complications	2	0	*n.s.*

In the Initial Phase (first 20 cases), representing the early adoption period, extensive soft-tissue dissection was performed until the lamina–facet junction and base of the spinous process were clearly visualized endoscopically before initiating bony drilling. In the Advanced Phase (subsequent 13 cases), after the surgical workflow was standardized, the extent of soft-tissue removal was deliberately limited, and drilling was started earlier once the laminar outline could be confidently identified using a combination of fluoroscopic guidance and direct endoscopic visualization of key bony landmarks. It is important to emphasize that in both phases, neural decompression was strictly performed under direct endoscopic visualization, with fluoroscopy serving only as an adjunct for level confirmation and general orientation.

Inclusion criteria comprised symptomatic single-level lumbar stenosis requiring surgical decompression and a minimum postoperative follow-up duration of 6 months. Exclusion criteria encompassed multilevel decompressions, previous lumbar spine surgery, and a follow-up period of less than 6 months.

To contextualize the learning curve, it is noted that at the time AFESS was introduced, the primary surgeon had extensive experience with uniportal endoscopic procedures, including approximately 170 cases of Full-Endoscopic Lumbar (FEL) decompression [18] and 450 cases of Interlaminar Endoscopic Lumbar Discectomy (IELD) (Ono et al., 2022). However, the surgeon had no prior experience with Unilateral Biportal Endoscopic (UBE) procedures. This background suggests that while the surgeon was highly proficient in endoscopic visualization and interlaminar anatomy, the specific bimanual triangulation mechanics required for AFESS represented a new technical skill set acquired during this series.

This study was approved by the institutional review board (IRB #B-2023-815), and written informed consent was obtained from all participants.

**Fig. 1:**
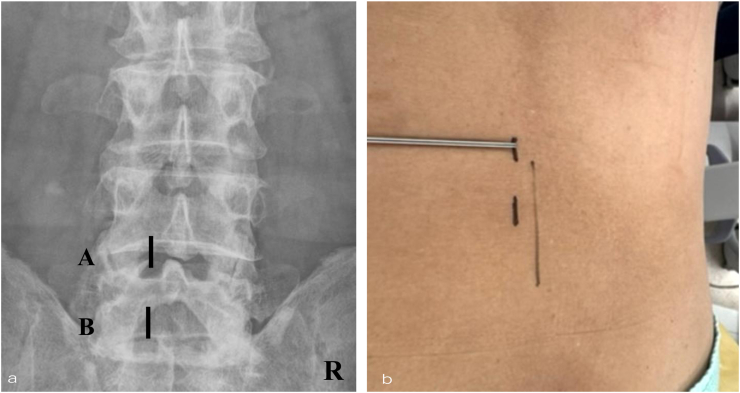
Portal Placement for AFESS (a) Fluoroscopic **anteroposterior** view showing the positioning of the camera portal (A) and the working portal (B). The working portal is located 2–3 cm caudal to the camera portal to establish a triangulated **working corridor**. (b) External photograph of the skin incisions. The two portals are spaced approximately 2–3 cm apart, allowing independent instrument manipulation. R = right side.

Surgical Technique (Advanced Phase) All procedures were performed under general anesthesia with patients positioned prone on a radiolucent operating table. The surgical steps described below correspond to the refined AFESS technique used in the advanced phase. Using fluoroscopic guidance, two small skin incisions (each <1 cm) were marked approximately 2 cm apart at the target lumbar level to establish separate camera and working portals (Fig. 1). The camera portal was positioned at the inferior border of the superior lamina (Fig. 1a, point A), while the working portal was situated 2 cm caudally (Fig. 1a, point B). After subcutaneous infiltration with an epinephrine-containing local anesthetic, sequential skin and fascial incisions were made. A pencil dilator followed by a bevel-type cannula was advanced toward the lamina under fluoroscopic visualization. Subsequently, a full-endoscope, a 25-degree rigid spinal endoscope (RIWOspine GmbH, Knittlingen, Germany) was introduced via the camera portal under continuous saline irrigation at a flow rate of 30–50 mL/min (Yeung, 2022a).

In the Advanced Phase, to optimize procedural efficiency, the initial bony resection was guided by a combination of fluoroscopic orientation and the surgeon's tactile feedback of the high-speed drill tip against the bony surface dorsal to the ligamentum flavum. This maneuver was facilitated by the surgeon's extensive prior experience with endoscopic anatomy (FEL/IELD). However, we emphasize that for surgeons in the learning phase or those with less endoscopic experience, maintaining strict direct visual identification of anatomical landmarks prior to drilling is the safest approach. Regardless of the drilling initiation method, the final neural decompression and removal of the deep layer of the ligamentum flavum were invariably performed under direct, continuous endoscopic visualization to ensure the safety of the neural elements.

**Fig. 2:**
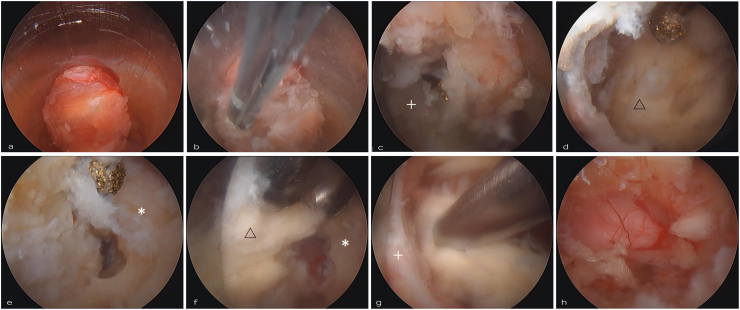
Ipsilateral Decompression Sequence (Advanced Phase) Step-by-step endoscopic views of the ipsilateral decompression. (a) Initial view of the paraspinal muscles after inserting the endoscope. (b) Targeted soft-tissue ablation using a radiofrequency bipolar probe, minimizing exposure to only the necessary bony landmarks. (c) Identification of the inferior margin of the superior lamina (+). The drill is introduced through the separate working portal. (d) Drilling of the superior lamina exposes the cranial attachment of the ligamentum flavum (△). (e) Drilling of the caudal lamina (∗) releases the inferior attachment of the ligamentum flavum. (f) Removal of the ligamentum flavum (△) using standard surgical forceps introduced through the working portal. (g) View after resection of the cranial portion of the ligamentum flavum. (h) Final endoscopic view showing the decompressed dural sac and traversing nerve root after ipsilateral decompression.

The initial endoscopic view revealed the paraspinal musculature (Fig. 2a). Under direct endoscopic visualization, the soft tissues were meticulously ablated using the Trigger-Flex Bipolar System (Elliquence, Baldwin, NY, USA) or TipControl® RF instruments (Richard Wolf GmbH, Knittlingen, Germany) and precision forceps, thereby exposing the inferior margin of the superior lamina (Fig. 2b and c) (Yeung, 2022b). Maintaining optimal distance between the cannula tip and endoscope facilitated visualization of tissue planes and minimized hemorrhage during controlled soft-tissue ablation (Fig. 2b).

After definitive verification of bony landmarks, high-speed drilling (Midas Rex MR8, Medtronic, Minneapolis, MN, USA) at 30,000–45,000 rpm was performed, initially targeting the base of the spinous process and the inferior border of the superior lamina (Fig. 2c) (Lee et al., 2023). The lamina–facet junction, medial aspect of the inferior articular process, and base of the spinous process were used as primary bony reference points during this step. Progressive, controlled drilling created adequate working space, clearly exposing the cranial (Fig. 2d) and caudal (Fig. 2e) attachments of the ligamentum flavum. Meticulous attention was given to limiting drilling depth to prevent inadvertent dural injury.

**Fig. 3:**
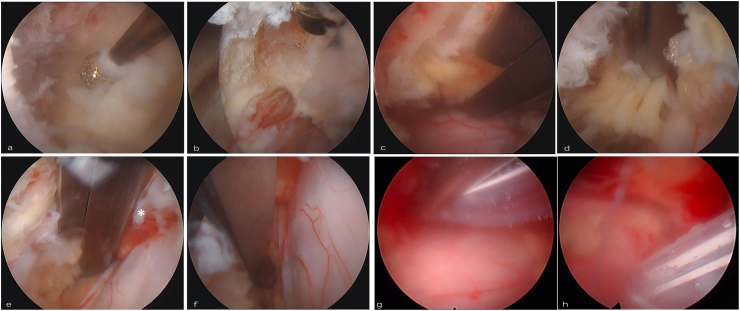
Technical Sequence of Contralateral Decompression Endoscopic views during the contralateral decompression phase. (a) Drilling of the contralateral cranial lamina to detach the ligamentum flavum. (b) Drilling of the contralateral caudal lamina. (c, d) Piece-by-piece resection of the adherent contralateral ligamentum flavum using a Kerrison rongeur introduced via the working portal. (e) Final lateral dissection exposing the contralateral dura and traversing nerve root, confirming sufficient decompression. (f) Pedicle-to-pedicle decompression is verified using a blunt dissector. (g, h) A 10 Fr surgical drain is inserted retrogradely through the working portal under direct endoscopic vision.

Ligamentum flavum resection was executed using standard surgical forceps and Kerrison rongeurs (Fig. 2f–h). The use of conventional microsurgical instruments through the working portal allowed familiar handling and mechanical leverage similar to that in open or microscopic decompression, while maintaining the advantages of an endoscopic approach. Following the completion of ipsilateral decompression, contralateral decompression was initiated with drilling of the contralateral cranial (Fig. 3a) and caudal lamina (Fig. 3b), employing principles analogous to those utilized ipsilaterally. Adherent contralateral ligamentum flavum was systematically removed using 2–3 mm Kerrison rongeurs (Fig. 3c and d) (Du et al., 2024). Lateral dissection adequately exposed the contralateral dura and nerve roots, confirming successful decompression (Fig. 3e). Complete bilateral decompression from pedicle to pedicle was verified under direct endoscopic visualization using a blunt dissector (Fig. 3f). Finally, a 10 Fr surgical drain was inserted retrogradely through the working portal under endoscopic guidance, with accurate placement visually confirmed before layered skin closure using 3-0 absorbable sutures (Fig. 3g and h).

Distinction from Conventional Unilateral Biportal Endoscopic Surgery In contrast to conventional unilateral biportal endoscopy, in which the endoscope and instruments can be exchanged between portals, AFESS maintains the full endoscope within a cannulated camera portal and uses the caudal working portal for standard spinal instruments throughout the procedure. This configuration provides a stable endoscopic view with continuous irrigation, while permitting bimanual manipulation and triangulation similar to biportal surgery and allowing the use of familiar microsurgical drills and rongeurs rather than long, dedicated endoscopic instruments.

**Fig. 4:**
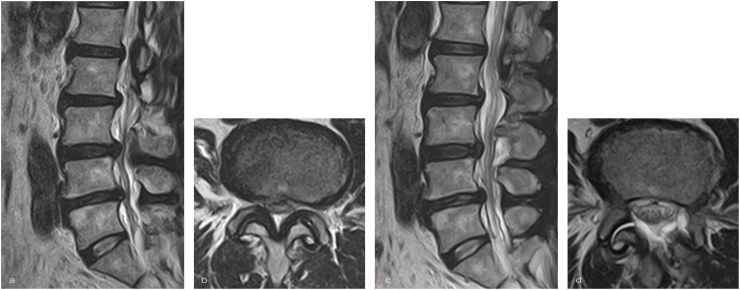
Radiological OutcomesPre- and postoperative magnetic resonance imaging (MRI) of a representative case. (a) Preoperative sagittal T2-weighted MRI showing severe spinal canal stenosis. (b) Preoperative axial T2-weighted MRI at the stenotic level. (c) Postoperative sagittal T2-weighted MRI demonstrating effective decompression. (d) Postoperative axial T2-weighted MRI showing expansion of the dural sac and restoration of the spinal canal area.

**Fig. 5:**
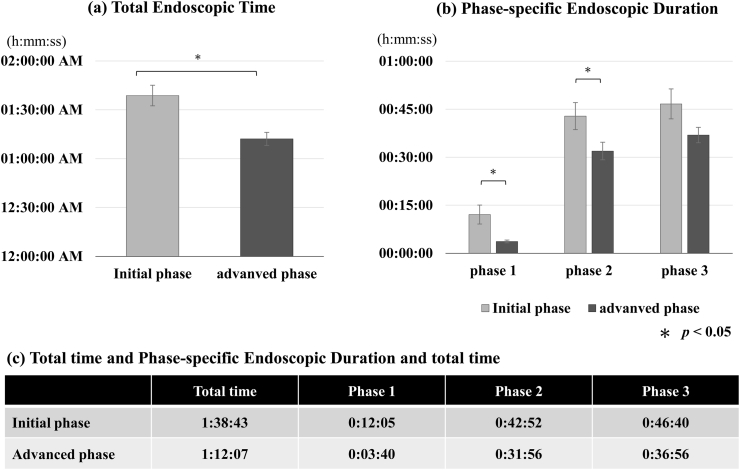
Operative Time Analysis Comparison of operative times between the initial and advanced phases. (a) Total operative time was significantly shorter in the advanced phase (p < 0.05). (b) Phase-specific duration analysis. Phase 1 (preparation): endoscope insertion to initiation of drilling. Phase 2 (approach): drilling to identification of the dura. Phase 3 (decompression): dural identification to procedure completion. Significant reductions were observed in Phase 1 and Phase 2 in the advanced phase. (c) Table presenting the detailed mean operative times for each phase. Data are presented as mean ± standard error. ∗ Indicates p < 0.05.

Postoperative Imaging and Outcome Assessment All patients underwent preoperative and 1-month postoperative lumbar magnetic resonance imaging (MRI) using a 1.5T scanner with T1-and T2-weighted sequences in both axial and sagittal planes to evaluate the efficacy of decompression (Fig. 4) (Choi et al., 2023). Clinical outcomes were comprehensively assessed, including operative duration, which was subdivided into three distinct phases: Phase 1 (endoscope insertion to drilling initiation), Phase 2 (drilling to dural identification), and Phase 3 (dural identification to procedure completion) (Fig. 5). These procedural intervals were precisely measured by independent reviewers through analysis of the intraoperative endoscopic video recordings to objectively evaluate procedural efficiency. All procedures were digitally recorded in high definition (1080p) for subsequent analysis. Postoperative hospitalization duration was also documented (Table 1). Estimated intraoperative blood loss was extracted from the anesthetic record for each case.

**Fig. 6:**
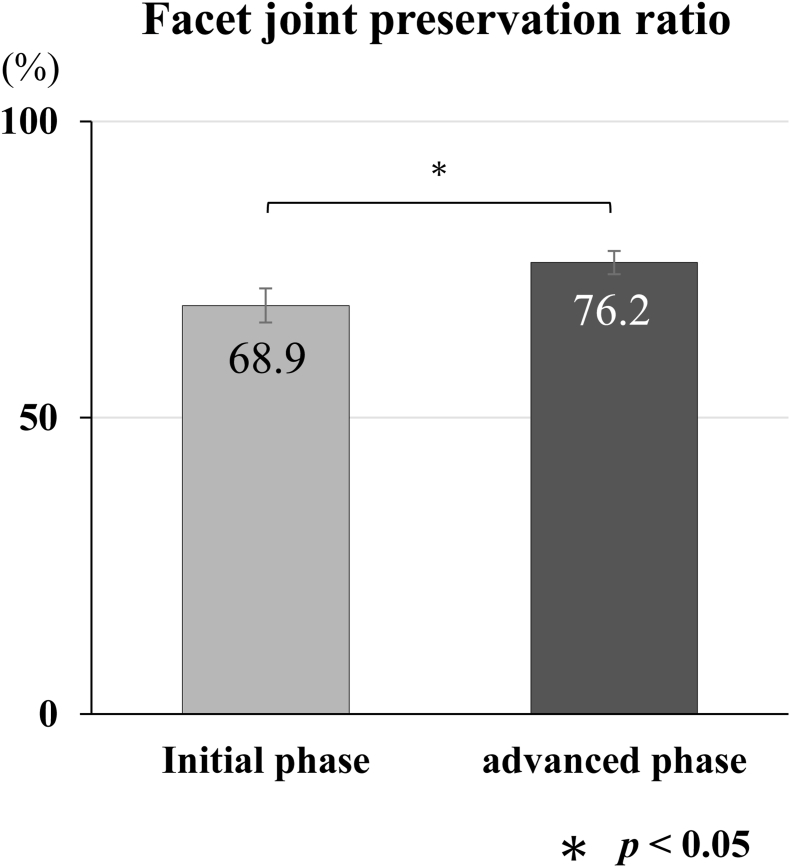
Facet Joint Preservation Comparison of the facet joint preservation ratio between the initial and advanced phases. The advanced phase (76.2 %) achieved a significantly higher preservation rate compared to the initial phase (68.9 %). **Data are presented as mean percentages; ∗ indicates p < 0.05.**

**Fig. 7:**
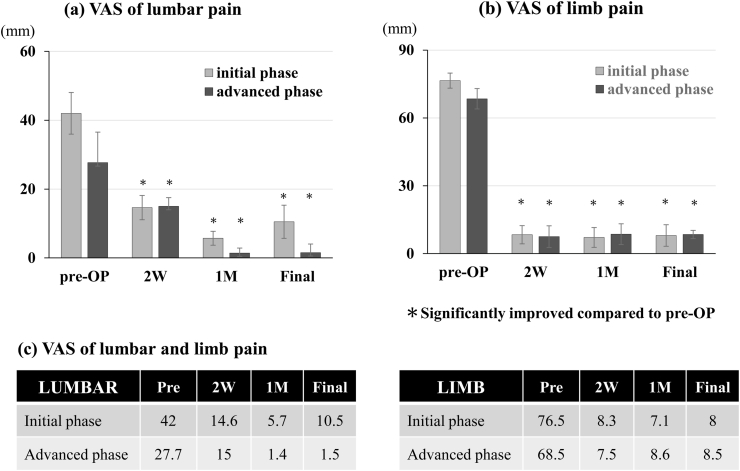
Pain Outcomes Following AFESS Visual analog scale (VAS) scores (0–100 mm) for (a) lumbar pain and (b) limb (radicular) pain. Both the initial-phase (gray bars/lines) and advanced-phase (black bars/lines) groups demonstrated significant improvement in pain scores at all postoperative time points compared with preoperative values. There was no statistically significant difference in pain relief between the two groups. **(c)**.

The facet joint preservation ratio, defined as the proportion of intact facet joints postoperatively relative to preoperative conditions, was quantitatively calculated using postoperative coronal computed tomography (CT) scans with 1-mm slice thickness (Fig. 6) (Tawfik et al., 2022). Pain outcomes were evaluated using a validated visual analog scale (VAS) for both lumbar and limb pain, assessed preoperatively and at postoperative intervals of 2 weeks, 1 month, and final follow-up (minimum 6 months) (Fig. 7). VAS scores were recorded on a 0–100-mm scale, with 0 indicating no pain and 100 indicating the worst imaginable pain. All complications were systematically documented, although the primary study endpoints were procedural duration, facet joint preservation ratio, and pain outcomes.

Statistical Analysis Data were presented as mean ± standard error for continuous variables and as frequencies with percentages for categorical variables. Differences in outcomes between initial-phase and advanced-phase groups were analyzed using independent-samples Student's t-tests for continuous variables and chi-square or Fisher's exact tests for categorical variables. Repeated-measures analysis of variance (ANOVA) was used to assess changes in VAS scores over time (Matsumura et al., 2010). Statistical significance was defined as p < 0.05. All statistical analyses were performed using SPSS version 25.0 (IBM Corp., Armonk, NY, USA) (Johnson and Thompson, 2022). A post-hoc power analysis was conducted to validate the adequacy of the sample size.

## 3 Results

### 3.1 Operative time

Intraoperative phase timing analysis demonstrated that the advanced phase group achieved significantly shorter operative durations compared to the initial phase group (100 min 12 s vs 72 min 7 s) (Fig. 5a). During Phase 1, the mean operative time decreased from 12 min 5 s in the initial phase to 3 min 40 s in the advanced phase (p = 0.011) (Fig. 5b). Similarly, the Phase 2 duration was significantly reduced in the advanced group (31 min 56 s vs 42 min 52 s; p = 0.038) (Fig. 5b). Phase 3 also showed a reduction from 46 min 40 s (initial) to 36 min 56 s (advanced), though this difference did not reach statistical significance (p = 0.075) (Fig. 5b).

### 3.2 Facet joint preservation

Facet joint preservation rates were high in both groups, with a higher preservation rate observed in advanced-phase surgeries. The overall facet joint preservation rate was 68.9 % in the initial-phase group and 76.2 % in the advanced-phase group (Fig. 6). This improvement reached statistical significance (p = 0.047).

### 3.3 Complications

One patient in the initial phase group developed a symptomatic hematoma that required surgical evacuation. Additionally, another patient in the initial phase group experienced an intraoperative dural tear that necessitated open conversion for dural repair. Thus, the overall complication rate in the initial phase group was 10.0 % (2/20), whereas no complications were observed among the 13 patients in the advanced-phase group (0/13). Fisher's exact test revealed no statistically significant difference in complication rates between the two groups (p = 0.51), and the small number of events precludes any firm conclusions regarding a lower complication risk in the advanced phase. The observed numerical reduction in complications with increasing case numbers is therefore presented as a descriptive finding.

#### 3.3.1 Pain outcomes

Both cohorts demonstrated significant improvements in pain following surgery (Fig. 7). For lumbar pain, the initial phase cohort's mean VAS improved from 42 mm preoperatively to 10.5 mm at the final follow-up (p < 0.01) (Fig. 7a). The advanced-phase cohort also showed improvement, with the mean lumbar VAS decreasing from 27.7 mm at baseline to 1.5 mm at final follow-up (Fig. 7a). Regarding radicular pain, both groups experienced substantial relief, with limb pain VAS decreasing from 77 to 8 mm (initial group) and from 68.5 to 8.5 mm (advanced group) at the final follow-up. A reduction in limb pain was observed in 89.1 % (initial group) and 86.8 % (advanced group), respectively, with no significant between-group differences in the timing or magnitude of radicular pain relief. All within-group improvements in both lumbar and limb pain were statistically significant at all postoperative time points compared with baseline (p < 0.05).

### 3.4 Estimated blood loss and hospital stay

Accurate quantification of intraoperative blood loss was challenging due to the continuous saline irrigation system inherent to the endoscopic procedure, which results in the immediate dilution of blood. Consequently, blood loss was recorded as “minimal” in the anesthetic records for all cases. Intraoperative bleeding was well-controlled and did not compromise the surgical field or procedural safety. No significant hemorrhage occurred in any case, and no patients required perioperative blood transfusion. The length of hospital stay was significantly shorter in the advanced phase group compared to the initial phase group (p < 0.05) (Table 1).

## 4 Discussion

### 4.1 Technical concept and positioning of AFESS

The present study describes Assisted Full-Endoscopic Spine Surgery (AFESS) as a technical modification of the unilateral biportal endoscopic (UBE) approach. While conventional UBE utilizes an arthroscope, AFESS incorporates a standard full-endoscopic system into the camera portal while maintaining a separate working portal for instrument manipulation. This configuration is not intended to replace established uniportal or biportal techniques but rather to offer an alternative option that leverages the high-definition optics and continuous irrigation system familiar to full-endoscopic surgeons, combined with the independent instrument triangulation characteristic of biportal surgery. Our experience suggests that for surgeons transitioning from uniportal full-endoscopic lumbar (FEL) surgery, AFESS may provide a more familiar visual field while overcoming the restriction of coaxial instrument movement. Conversely, for those accustomed to microscopic or open surgery, the bimanual workflow of AFESS may feel more intuitive than the single-port handling of FEL. However, compared with the single-incision approach of uniportal FESS, AFESS requires two incisions and involves a broader area of soft-tissue dissection to establish the triangulated working corridor. While this represents a higher degree of invasiveness than pure uniportal techniques, we believe the ergonomic benefits of independent instrumentation balance this drawback, particularly for complex pathologies.

## 5 Structural preservation and adequacy of decompression

A critical concern in minimally invasive spine surgery is whether limited bony resection compromises the adequacy of neural decompression. In our series, the facet joint preservation rate significantly improved from 68.9 % in the initial phase to 76.2 % in the advanced phase. It is important to clarify that this high preservation rate does not imply insufficient decompression. Rather, it reflects the advantage of the endoscopic visual angle, which allows for “under-cutting” of the facet joint (IBM Corp, 2017). By placing the endoscope within the spinal canal, the surgeon can visually inspect the lateral recess and neuroforamen from the inside out. This perspective enables the selective removal of compressing factors—such as the hypertrophied ligamentum flavum and osteophytes—while sparing the dorsal load-bearing portion of the facet joint (Kaen et al., 2023). Therefore, the preservation of posterior stabilizing structures in AFESS is achieved through targeted, inside-out decompression rather than by limiting the necessary extent of surgical release. The significant improvement in leg pain scores and the radiological confirmation of canal restoration in our series support the efficacy of this selective approach.

## 6 Clinical outcomes and efficacy

Consistent with the radiological findings, clinical outcomes demonstrated significant pain relief in both cohorts. The substantial reduction in VAS scores for both lumbar and radicular pain was comparable to outcomes reported for established endoscopic techniques (Yang et al., 2023; Fogel et al., 2019). Notably, there was no statistically significant difference in pain relief between the initial and advanced phases. This finding indicates that while the standardization of the technique in the advanced phase significantly improved operative efficiency (reduced operative time), the fundamental efficacy of neural decompression was successfully achieved even during the early adoption phase. This suggests that the learning curve for AFESS primarily affects surgical speed rather than the quality of clinical outcomes.

### 6.1 Interpretation of the learning curve

Our analysis demonstrated a significant reduction in operative time after the first 20 cases, suggesting a rapid learning curve for this technique. However, this finding must be interpreted in the context of the surgeon's background. The primary surgeon in this study had extensive prior experience with uniportal endoscopic procedures, including approximately 170 cases of FEL and 450 cases of Interlaminar Endoscopic Lumbar Discectomy (IELD). This proficiency with endoscopic anatomy and image orientation likely accelerated the acquisition of AFESS skills, as the primary challenge was adapting to bimanual triangulation rather than interpreting the endoscopic view. Consequently, the learning curve of approximately 20 cases observed in this study, which is shorter than the 50 cases often cited as required to master conventional full-endoscopic techniques (Yang et al., 2022), may represent a “best-case scenario” for experienced endoscopists. For surgeons without prior endoscopic experience, the learning curve for AFESS would likely be longer and steeper. Future studies involving surgeons with varying levels of expertise are needed to establish generalizable training guidelines (Heo and Kim, 2021).

### 6.2 Safety profile and complication analysis

In this early single-surgeon series, AFESS was associated with a low complication rate: 2 events occurred, both in the initial phase (one symptomatic postoperative hematoma requiring evacuation and one intraoperative dural tear requiring open repair), whereas no complications were observed in the 13 advanced-phase cases. Overall, the complication rate was 6.1 % (2/33), and the difference between the initial and advanced phases (10.0 % vs 0 %) was not statistically significant (Fisher's exact test, p = 0.51). Given the small sample size and limited number of events, these data should be interpreted descriptively rather than as evidence that complications can be avoided after a certain number of cases.

Nevertheless, the absence of complications in the advanced phase may be clinically relevant and could be attributed to the stabilization of the surgical workflow. Separating the camera and working instruments in AFESS reduces instrument conflict within the spinal canal, potentially lowering the risk of incidental dural injury compared with uniportal techniques, in which instruments share the same axis (Yang et al., 2022). Furthermore, the improved fluid management and the ability to use independent bipolar cautery probes likely contributed to a safer operative environment. The overall complication profile in this series appears comparable to that reported for other endoscopic decompression procedures for LSS, including a 2–7 % incidence of dural tears in larger series (Yang et al., 2022). While these initial results are promising, larger, prospective comparative studies are required to determine whether the bimanual configuration of AFESS translates into an actual, reproducible reduction in complication rates.

## 7 Limitations

Several limitations of this study should be acknowledged. First, it is a retrospective analysis of a small case series from a single institution, which limits the statistical power and generalizability of the findings. Second, the follow-up period is relatively short; long-term data are required to assess the durability of decompression and the clinical significance of facet preservation regarding spinal instability. Third, as discussed, the learning curve data is specific to a surgeon with high preexisting endoscopic proficiency. Finally, this study did not include a control group treated with conventional FEL or UBE, precluding direct comparative conclusions. Future prospective multicenter studies are warranted to validate the relative benefits of AFESS compared with established minimally invasive techniques.

## 8 Conclusion

AFESS is a feasible, that allows effective neural decompression while preserving the facet joints. The technique permits bimanual instrumentation under high-quality endoscopic visualization. Our early experience indicates that with appropriate training and standardization, AFESS can be performed safely and efficiently, particularly by surgeons with prior endoscopic experience. Further research is necessary to define its precise indications and long-term advantages over existing methods (Hofstetter et al., 2020).

Demographic and clinical data of 33 patients undergoing AFESS for lumbar spinal stenosis. **The initial-phase group (n** = **20) and advanced-phase group (n** = **13) are presented separately.** Data are presented as mean (range) for continuous variables and as number of cases for categorical variables. Estimated blood loss was recorded as “Minimal” in all cases due to the difficulty of accurate quantification under continuous saline irrigation. **The advanced-phase group had significantly shorter postoperative hospital stays (p** = **0.026) and shorter follow-up periods, reflecting the chronological nature of the series (p < 0.01).** n.s. = not significant.

## Funding statement

This study received no funding.

## Conflict of interest

The authors declare that they have no known competing financial interests or personal relationships that could have appeared to influence the work reported in this paper. None of the authors have any proprietary interests or conflicts of interest related to this submission. No financial support or funding was received for this study. This research did not receive any specific grant from funding agencies in the public, commercial, or not-for-profit sectors.

## Data Availability

Data used to support the findings of this study are available from the corresponding author upon reasonable request.
